# Characterisation of *CYP2C8*, *CYP2C9 *and *CYP2C19 *polymorphisms in a Ghanaian population

**DOI:** 10.1186/1471-2350-10-124

**Published:** 2009-12-02

**Authors:** William Kudzi, Alexander NO Dodoo, Jeremy J Mills

**Affiliations:** 1 Schools of Pharmacy and Biomedical Sciences, University of Portsmouth, St. Michael's Building, White Swan Road, Portsmouth PO1 2DT, UK; 2 Centre for Tropical Clinical Pharmacology and Therapeutics, University of Ghana Medical School. P.O. GP 4236, Accra, Ghana

## Abstract

**Background:**

Genetic influences on drug efficacy and tolerability are now widely known. Pharmacogenetics has thus become an expanding field with great potential for improving drug efficacy and reducing toxicity. Many pharmacologically-relevant polymorphisms do show variability among different populations. Knowledge of allelic frequency distribution within specified populations can be useful in explaining therapeutic failures, identifying potential risk groups for adverse drug reactions (ADRs) and optimising doses for therapeutic efficacy. We sought to determine the prevalence of clinically relevant Cytochrome P450 (*CYP) 2C8*, *CYP2C9*, and *CYP2C19 *variants in Ghanaians. We compared the data with other ethnic groups and further investigated intra country differences within the Ghanaian population to determine its value to pharmacogenetics studies.

**Methods:**

RFLP assays were used to genotype *CYP2C8 *(*2, *3, *4) variant alleles in 204 unrelated Ghanaians. CYP2C9*2 and *CYP2C19 *(*2 and *3) variants were determined by single-tube tetra-primer assays while *CYP2C9 *(*3, *4, *5 and *11) variants were assessed by direct sequencing.

**Results:**

Allelic frequencies were obtained for CYP2C8*2 (17%), CYP2C8*3 (0%), CYP2C8*4 (0%), CYP2C9*2 (0%), CYP2C9*3 (0%), CYP2C9*4 (0%), CYP2C9*5 (0%), CYP2C9*11 (2%), CYP2C19*2 (6%) and CYP2C19*3 (0%).

**Conclusion:**

Allele frequency distributions for *CYP2C8*, *CYP2C9 *and *CYP2C19 *among the Ghanaian population are comparable to other African ethnic groups but significantly differ from Caucasian and Asian populations. Variant allele frequencies for *CYP2C9 *and *CYP2C19 *are reported for the first time among indigenous Ghanaian population.

## Background

Variant allele frequencies of many pharmacogenetically-relevant polymorphisms have been demonstrated to vary greatly between populations of different countries. However, some areas of the world especially indigenous African populations have scarcity information in the current pharmacogenetics research [1,2] Cytochrome P450 2C (CYP2C) subfamily of enzymes form 18-30% of human CYPs and metabolises nearly 20% of all therapeutic drugs commonly prescribed in clinical practice [3]. *CYP2C *gene is made up of four isoforms, *CYP2C8*, *CYP2C9*, *CYP2C18 *and *CYP2C19 *which are located together on chromosome 10q24. This CYP2C subfamily of enzymes constitutes 15-20% of the CYP protein in the liver [3] and exhibit genetic polymorphisms leading to differences in activities of these enzymes. Genetic polymorphisms of this CYP2C subfamily of enzymes are thought to influence both efficacy of drugs and the likelihood of ADRs [4].

*CYP2C8*, *CYP2C9*, *and CYP2C19 *enzymes constitute 26%, 50%, and 16% respectively of the CYP2C subfamily [5]. They are polymorphically expressed with variable allele frequencies among different ethnic populations [2,6,7], Some of these CYP2C variant alleles have been associated with either an increased or decreased metabolism of several clinically important drugs [3,5,8,9]. Some of these drugs have narrow therapeutic index and have been involved in the development of adverse side effects. Variant allele frequencies in different populations do have implications for adverse effects. Patients carrying either the homozygous or the heterozygous variant alleles of CYP2C9 are at risk of acute gastrointestinal bleeding when treated with NSAID that are substrates of CYP2C9 [10]. CYP2C9 polymorphisms have been associated with an increase risk of bleeding in patients treated with standard doses of warfarin [11] while phenytoin toxicity has also been reported in some patients [12]. The U.S. FDA has added genetic information to prescribing information on some of these drugs such as Warfarin, carbamazepine and codeine to assist prescribers safely achieve therapeutic doses http://www.fda.gov/Drugs/ScienceResearch/ResearchAreas/Pharmacogenetics/ucm083378.htm.

Our aim is to genotype pharmacologically-relevant CYP2C8, CYP2C9 and CYP2C19 variants in normal Ghanaian population to address the lack of data in indigenous African population. Allele frequencies of major Ghanaian tribes were also compared to determine the genetic variation within the country. Allele frequencies were also compared with previously reported frequencies in published literature to determine inter ethnic variation.

## Methods

### Subjects

This study population was comprised of 204 healthy, unrelated individuals (92 males, 114 Females) from Accra, Ghana. Ethical approval for the study was obtained from the University of Portsmouth and the University of Ghana Medical School local research ethics committees. Written informed consents were obtained from all subjects prior to inclusion in the study. Blood samples (1 mL) were collected from the Ghanaian volunteers using EDTA anticoagulant tubes and 500 μL aliquots of the blood sample was spotted and preserved on coded Whatman FTA cards (Fisher Scientific, UK). The cards were placed in plastic bags and shipped to University of Portsmouth (UK) for analysis.

### DNA samples

Genomic DNA was extracted from 2 mm discs cored from blood spots following the manufacturer's protocol. Briefly, the discs were incubated for 5 min in 200 μL FTA purification reagent after which the used reagent was discarded. This process was repeated three times in total for FTA reagent and two times for buffer TE. The discs were then dried at 56°C for 10 min [13].

### Genotyping

CYP2C8*2 (805A > T), CYP2C8*3 (416G > A and 1196A > G) and CYP2C8*4 (792C > G) variants were identified by polymerase chain reaction-restriction fragment length polymorphism (PCR - RFLP) as described previously by Dai et al and Bahadur et al [6,14].

The presence of CYP2C9*2 variant allele was determined by single-tube tetra-primer PCR assay previously described by Allabi *et al *[15]. Identification of CYP2C9*3, CYP2C9*4, CYP2C9*5 and CYP2C9*11 variants were performed by direct sequence analysis of a single PCR product which amplified the *CYP2C9 *exon 7 [15]. CYP2C19*2 and CYP2C19*3 variant alleles were determined by single-tube tetra-primer PCR assay previously described by Hersberger *et al *[16].

Intra-country genetic variation was also determined the major tribes within the Ghanaian population. The tribes (Akan, Fanti, Ewe, Northern, and Ga) were determined by self reporting by the individual members of the study population. The samples were separately analysed for each tribe to find out if there were any genetic differences. Allele frequencies were compared with data previously reported for other African countries. Additionally, our data was compared with the highest and the lowest allele frequency values for Caucasian and Asian populations.

### Statistical analysis

Allele and genotype frequencies of SNPs analysed were determined by population genetic analysis program SNPAlyze version 5.1 (Dynacom Co. Ltd., Yokohama, Japan). Genotype deviations from the Hardy-Weinberg equilibrium were also determined. Linkage disequilibrium (LD) of all possible two-way combinations of SNP was tested using r^2^. Allele frequencies were compared with published data using Fisher's Exact Test. P-value ≤ 0.05 was considered significant.

## Results and Discussion

Allele and genotype frequencies for all polymorphisms screened are summarized in Table 1.

**Table 1:** Allele and Genotype frequencies for *CYP2C8*, *CYP2C9 *and *CYP2C19 *alleles observed in the Ghanaian population (%, 95% CI in parenthesis)

	CYP2C8*2	CYP2C9*11	CYP2C19*2
Total no. genotyped	203	195	169
Major allele homozygous	139 (68, 62.0 - 74.86)	190 (97.5, 95.22 - 99.66)	152 (90, 85.48 - 94.52)
Heterozygous	59 (29, 22.8 - 35.31)	4 (2, 0.06 - 4.04)	14 (8, 3.91, 12.09)
Minor allele homozygous	5 (3, 0.33 - 4.59)	1 (0.5, -0.49 - 1.51)	3 (2, -0.21, 3.77)
			
Major allele frequency	83 (79.35 - 86.65)	98 (96.61- 99.39)	94 (91.47- 96.53)
Minor allele frequency	17(13.35 - 20.65)	2 (0.61, 3.39)	6 (3.47, 8.53)

CI, Confidence Interval. CYP2C8*3, *4, CYP2C9*2, *3 *4, *5 and CYP2C19*3 variant alleles were not detected in this study

A total of 204 samples were collected for analysis, however, between 169 and 204 were available for each SNP. All the genotypes were in Hardy-Weinberg equilibrium. The pairwise LD between each SNP analysed was performed and the results expressed in Table 2. Three SNPs, CYP2C9*3, CYP2C9*4 and CYP2C9*5 showed a strong linkage disequilibrium. The major allele was defined as the most commonly occurring allele in the population. CYP2C8*2 variant was the most frequent allele detected at 17%. CYP2C8*3 and CYP2C8*4 variant alleles were not detected in this study. Homozygous variant alleles were detected in 3% of the study population while 29% of individuals carried at least one variant allele (Table 1). CYP2C9*2, CYP2C9*3, CYP2C9*4 and CYP2C9*5 variant alleles were not detected in the present study. CYP2C9*11 variant allele frequency was 2% (Table 1). Homozygous CYP2C9*11 variant was found in one individual, while four individuals were heterozygous for the CYP2C9*11 variant (Table 1). Carriers of the CYP2C19*2 variant allele were found in twenty individuals (6%) in this study population; three of these individuals were homozygous for the CYP2C19*2 variant while 14 individuals were heterozygous for CYP2C19*2 variant. The CYP2C19*3 variant was not detected in the study population.

**Table 2:** Analysis of linkage disequilibrium for a set of 10 SNPs in CYP2C locus in a Ghanaian population.

	CYP2C8*2	CYP2C8*3	CYP2C8*4	CYP2C9*2	CYP2C9*3	CYP2C9*4	CYP2C9*5	CYP2C9*11	CYP2C19*2	CYP2C19*3
CYP2C8*2										
CYP2C8*3	0									
CYP2C8*4	0	0								
CYP2C9*2	0.00001459	0	0							
CYP2C9*3	0.0008986	0	0	0.0402						
CYP2C9*4	0.0008986	0	0	0.0402	**1**					
CYP2C9*5	0.0008986	0	0	0.0402	**1**	**1**				
CYP2C9*11	0.001332	0	0	0.002015	0	0	0			
CYP2C19*2	0.0134	0	0	0.0209	0.003165	0.003165	0.003165	0.001274		
CYP2C19*3	0.00342	0	0	0.2237	0.1631	0.1631	0.1631	0.003538	0.0121	

Pairwise LD represented as r^2 ^(from 0 to 1) is expressed in each cell. SNPs with strong linkage disequilibrium are highlighted in gray

Intra-population variations were determined for five major tribes within the Ghanaian population were self-identified as Akan, Fanti, Ewe, Northern, and Ga tribes. Intra-population data was similar for all the SNPs analysed except for CYP2C8*2 variant which was significantly higher in the Northern tribe (p = 0.025). CYP2C9 *11 variant was detected in only the Ewe and Ga tribes at frequencies of 5% and 3% respectively. The Ghanaian population was found to a large extent to be genetically homogenous.

The current study investigated three previously described polymorphisms of the *CYP2C8 *and the data was compared with other studies reported in published literature (Table 3). The CYP2C8*2 variant allele was observed in 17% of this study population consistent with data from an earlier study among the Ghanaian population (17%) [17] and other populations of African origin such as African American (18%) [6,18], Burkina Faso (11.5%) [19] and Zanzibaris (14%) [20]. Conversely the CYP2C8*2 variant is very infrequent in the Caucasian populations (0-2%) [6,14,21,22] and absent in Asian populations [2]. The CYP2C8*3 and CYP2C8*4 variant alleles were not detected in this study population. These variant alleles have rarely been reported in other African populations [18-20,23]. CYP2C8*3 variant allele, however, has been reported between 9.5-17% within the Caucasian population [2]. The CYP2C8*4 variant allele is rare among Caucasian populations (0-7.5%). CYP2C8*3 and CYP2C8*4 variant alleles have not been reported within Asian populations [2]. The presence of CYP2C8*3 and CYP2C8*4 variant alleles in Caucasians differs significantly from data from the present study.

**Table 3:** Allele frequencies of *CYP2C8, CYP2C19 *and *CYP2C19 *variants in a Ghanaian and other previously studied populations

	*CYP2C8* *2	*CYP2C8* *3	*CYP2C8* *4	*CYP2C9* *2	*CYP2C9* *3	*CYP2C9* *4	*CYP2C9* *5	*CYP2C9* *11	*CYP2C19* *2	*CYP2C19* *3
**African**										
Ghanaian	0.17	0	0	0	0	0	0	0.02	0.06	0
Ghanaian	0.17 [23]	0 [23]	0 [23]							
Zanzibaris	0.14 [20]	0.02 [20]	0.006 [20]							
Burkinabe	0.115 [19]	0.004 [19]	0 [19]							
Beninese				0 [15]	0 [15]	0 [15]	0.02 [15]	0.03 [15]	0.13 [15]	0[15]
Ethiopian				0.043 [24]	0.023 [24]		0 [24]			
Zimbabweam									0.13 [34]	0[34]
African American	0.18 [6]	0.02 [6]		0.033 [18]	0.023 [18]	0 [18]	0.01 [18]	0.02 [18]	0.25 [33]	0 [33]
										
**Caucasian **§	0 - 0.004 [2]	0.095 - 0.17 [2]	0 - 0.075 [2]	0.08 - 0.191 [2]	0.03 - 0.17 [2]	0 - 0.004 [15]	0 [2]		0.09 [15] - 0.14 [42]	0
										
**Asian **§	0 [2]	0 [2]	0 [2]	0 - 0.001 [2]	0.011 - 0.068 [2]				0.23 [33] - 0.305 [43]	0.085 [43]

§ The lowest and the highest allele frequencies for Caucasian and Asian populations

CYP2C8*2 variant detected at 17% in this study and has a reduced potential of metabolising paclitaxel and arachidonic acid. The CYP2C8*2 variant allele results in 15% reduction in activity towards paclitaxel metabolism *in vitro *compared to the wild-type allele (CYP2C8*1) [6] while the CYP2C8*3 variant allele showed 50% reduction in activity to paclitaxel metabolism [14]. CYP2C8*2 and CYP2C8*3 variant genotypes can lead to poor metaboliser (PM) phenotype which could potentially cause drug toxicity. Although CYP2C8*2 and CYP2C8*3 variant alleles have been associated with reduced enzymatic activity only the CYP2C8*2 variant was detected in the current study.

Five pharmacologically relevant *CYP2C9 *variants were surveyed in the current study and the results compared with other studies (Table 3). The CYP2C9*2 and CYP2C9*3 variant alleles were not found in this study population and this was consistent with an earlier report in a Beninese population [15]. This observation, however, was different from frequencies obtained within the African American and Ethiopian populations. The CYP2C9*2 and CYP2C9*3 variant alleles were reported at 3.3% and 2.3% respectively in African American populations [18] and at 4.3% and 2.3% in the Ethiopian population [24]. In contrast, CYP2C9*2 variant allele has been reported at 8-19% and the CYP2C9*3 variant allele at 3.3-17% among the Caucasian population [2]. The CYP2C9*2 variant allele was rarely seen in Asian populations while the CYP2C9*3 variant allele was prevalent at 1.1-6.8% within the Asian populations [2].

The CYP2C9*5 variant allele was not detected in the current study but has been reported at a low level in Beninese (2%), but slightly higher than the Tanzanian (0.8%) and African American (1%) populations [15,18]. This variant allele is not reported among the Caucasian populations [2]. The CYP2C9*11 variant allele was detected at 2% frequency within this study population. This frequency was similar to data from Beninese (3%) and African American (2%) populations [15,18]. This variant allele was rare among the Caucasian [15] and was not reported in the Asian population.

CYP2C9*2 and CYP2C9*3 polymorphisms have been shown to cause a decrease in enzymatic activity of 30% and 80% respectively [25,26] and have been shown to have clinical implications for patients carrying these alleles [27]. The CYP2C9*3 variant allele has been reported to cause the largest reduction in metabolic capacity for many *CYP2C9 *substrates, followed by the CYP2C9*2 variant allele causing an intermediate reduction when compared to the wild-type allele (CYP2C9*1) [28]. Poor metabolisers, mostly CYP2C9*3/CYP2C9*3 carriers, may experience severe toxicity when metabolising *CYP2C9 *substrates with narrow therapeutic index such as warfarin and phenytoin. Conversely, these PMs may not have an adequate drug response and may experience therapeutic failure when taking a pro-drug such as losartan and cyclophosphamide, that requires bio-activation by *CYP2C9 *[21]. ADRs to CYP2C9 substrate drugs like warfarin and glipizide are more evident in African/Afro-Caribbean populations than Caucasians, however, CYP2C9*2 and CYP2C9*3 polymorphisms are not common in this ethnic group [29,30].

CYP2C19*2 and CYP2C19*3 variant alleles are the most characterised alleles of the *CYP2C19 *gene [31,32]. The CYP2C19*2 variant allele was the most common allele found in the current study as in other studies (Table 3). Significant inter-ethnic difference has been reported for CYP2C19*2 variant allele in the published literature [15,33]. It occurred at a lower frequency of 6% within this study population compared to 13% in the Beninese [15] and Zimbabwean (13%) populations [34]. CYP2C19*2 variant allele has been reported at similar frequencies in Caucasian populations (9%-14%) [33,35]. The prevalence of the CYP2C19*2 variant allele has been reported at significantly high frequencies within the African American (25%) and the Asian populations (23-30.5%) (p = 0.003; and p = 0.001, respectively) [2,36]. CYP2C19*3 variant allele was not detected in this study which is consistent with findings within other African populations [15,34]. Although the CYP2C19*3 variant has been rarely reported in Caucasian populations [15,33], it has been found in Asian populations at a frequency of 10% [33,37].

CYP2C19*2 variant detected in 6% of the study population were predicted to be PMs for *CYP2C19 *substrates and are potentially more prone to the possibility of suffering from adverse drug effects than extensive metabolisers (EMs) when taking therapeutic doses of drugs such as warfarin, diazepam and omeprazole. CYP2C8*5, CYP2C9*6 and major CYP2C18 gene variants were not analysed due to shortage of DNA sample.

Although the ultimate aim of pharmacogenetics is to lead to personalised treatment of individual patients based on their genetic profile, the identification of polymorphisms within populations can also be useful in improving the quality of healthcare in that specified population. Investigating the genetic variation within genes encoding drug metabolising enzymes (DMEs), drug transporters and drug targets within different populations is becoming increasing important because of the drug-drug interactions that results from enzyme inhibitions or inductions. This type of information is increasingly becoming useful for improving drug therapy and explaining inter-individual and inter-ethnic differences due to drug response [38,39]. It is also being used to predict and explain ADRs which cause 7% of all hospital admissions and 4% withdrawal of new medications [40,41]. It is becoming increasing important to derive data from different populations to build a database which can then be used in epidemiological investigations to better understand the genetic risk factors which affect many diseases and to be in a better position to predict them in the future.

## Conclusion

The current study has led to the determination of allelic variants of *CYP2C8*, *CYP2C9 *and *CYP2C19 *in a Ghanaian population. Some of these variants alleles to our knowledge are being reported for the first time among the Ghanaian population. The frequencies obtained are comparable to data previously reported in other populations of African origin but differ from that observed in Caucasian and Asian populations. These results provide additional information on polymorphisms of this CYP2C subfamily of enzymes in an indigenous African population which is scarce in published literature.

## Competing interests

The authors declare that they have no competing interests.

## Authors' contributions

The study was conceived and designed by WK and JJM. WK performed the experimental analysis and drafted the manuscript. The implementation was supervised by JJM. ANOD and JJM assisted in interpreting the results and drafting the manuscript. All authors read and approved the final manuscript.

## Pre-publication history

The pre-publication history for this paper can be accessed here:

http://www.biomedcentral.com/1471-2350/10/124/prepub
